# Transcriptome Analysis Reveals the Anti-Tumor Mechanism of Eucalyptol Treatment on Neuroblastoma Cell Line SH-SY5Y

**DOI:** 10.1007/s11064-022-03786-8

**Published:** 2022-11-04

**Authors:** Kai Gao, Congying Wu, Yanlong Li, Jian Lu, Yuwu Jiang

**Affiliations:** 1 Department of Pediatrics, Peking University First Hospital, No.1 Xi ’ an Men Street, West District, 100034 Beijing, China; 2 Department of acupuncture and moxibustion, Dongzhimen Hospital, Beijing University of Chinese Medicine, No.116 Cuiping West Street, Tongzhou District, 101121 Beijing, China

**Keywords:** Eucalyptol, 1.8-cineole, Moxibustion, Anti-cancer, *MYC*, RNAseq

## Abstract

Eucalyptol (1.8-cineole), an active component in traditional Chinese medicine *Artemisia argyi* for moxibustion. Previous studies have shown that eucalyptol has anti-tumor effects on leukemia and colon cancer. Nonetheless, the effect and mechanism of eucalyptol on neuroblastoma remains unclear. In the present study, we intended to reveal the effect and mechanism of eucalyptol treatment on the neuroblastoma cell line SH-SY5Y through transcriptome analysis. In the group treated with eucalyptol, 566 brain genes were up-regulated, while 757 genes were down-regulated. GO function analysis showed that positive regulation of cell cycle was down-regulated in biological processes. Meanwhile, cancer-related pathways were identified in KEGG (Kyoto Encyclopedia of Genes and Genomes) enrichment analysis, including pathways in cancer, PI3K-Akt signaling pathway, cAMP signaling pathway, TGF-beta signaling pathway, Hippo signaling pathway, p53 signaling pathway, and additional pathways. Furthermore, we found a key gene, such as *MYC*, by constructing a network of cancer related pathways with differentially expressed genes and transcription factor analysis. In conclusion, our research indicates that *MYC* might play a central role in the anit-tumor mechanisms of eucalyptol.

## Introduction

Neuroblastoma is a developmental tumor of children from the neural crest. This disease is the primary cause of cancer-related death in children under 5 years of age [1]. Neuroblastoma is a heterogeneous pediatric tumor. Half of this disease is a high risk type and lacks effective cures [2]. SH-SY5Y cell line is a subclone of human neuroblastoma, originally derived from a child metastatic bone tumor biopsy [3]. This cell line is a common cell model to study neurotoxicity [4], neurodegenerative disease [5], neuron differentiation and neuroblastoma [6].

Eucalyptol is a cyclic ether and monoterpenoid, naturally produced by plants such as *Artemisia argyi* [7, 8]. It is an ingredient in some brands cough suppressants and is also used as a flavoring agent [9]. Eucalyptol has been reported to possess multiple pharmacological effects, including anti-inflammatory [10–12], antioxidant effects [13], pain reduction [14, 15], epilepsy inhibition [16], et al. In some *in vitro* studies, eucalyptol has anti-tumor effect on leukemia [17], ovarian cancer cells [18] and colon cancer cell line [19]. However, the anti-tumor effect of eucalyptol on neuroblastoma is still unknown.

In this research, we investigated the anti-tumor mechanism of eucalyptol by transcriptome sequencing and bioinformatic analysis. And we discovered that eucalyptol exerts anti-tumor activity on human neuroblastoma cell lines SH-SY5Y by regulating several cancer related pathways and genes. Our findings will be valuable for understanding the anti-tumor mechanism of eucalyptol in neuroblastoma cell proliferation and provide a new therapeutic candidate agent for neuroblastoma therapy.

## Methods

### Cell Culture of SH-SY5Y

The SH-SY5Y was kindly given by Professor Yun Wang of the Neuroscience Research Institute, Peking University. Cell cultures of SH-SY5Y were cultured in DMEM/F12 (Sigma, Darmstadt, Germany) with 10% (v/v) fetal bovine serum (FBS) (Gibco, Grand Island, USA) and 1% Penicillin-Streptomycin Solution (Gibco, Grand Island, USA). All cultures were incubated in a Thermo CO_2_ incubator at 37℃ with 95% air and 5% CO_2_ (v/v) and a humidity of 95%. The cell culture medium was changed twice a week. 70%~80% of confluent cultures used for passage to experiments.

### Transcriptome Sequencing

Three pairs of cell samples were collected from untreated and 100 µM eucalyptol-treated SH-SY5Y cells for 6 days, and RNA was extracted for RNA-seq by Trizol (Invitrogen, Carlsbad, CA, USA). Two micrograms of RNA per sample were used as input material for the RNA sample preparations. Sequencing libraries were generated with the VAHTS mRNA-seq v2 Library Prep Kit for Illumina following the manufacturer’s recommendations. Index codes were added to attribute sequences to each sample. Then libraries were sequenced using an Illumina NovaSeq platform to generate 150 bp paired-end reads according to the manufacturer’s instructions.

Raw data of FASTQ format was processed first through primary quality control. In this step, clean data were obtained by removing read pairs that contain N more than 3 or the proportion of base with quality value below 5 is more than 20%, in any end, or adapter sequence was founded. The clean data of each sample was more than 6 GB. All the downstream analyses were based on clean data with high quality.

### Differential Expression Analysis and Venn Diagrams

Alignment of paired-end clean reads to the reference genome was with TopHat (v2.1.1). Differential expression analysis between two conditions was performed using Cufflinks (v2.2.1). Differently expressed genes (DEGs) were defined as those for which the *P*-value below 0.01 and the absolute value of log_2_(Fold change) more than 1. The Venn Diagrams were constructed by an interactive Venn diagram viewer [20].

### Functional Enrichment Analysis

GO and KEGG enrichment analysis of DEGs sets were executed by the Database for Annotation, Visualization and Integrated Discovery (DAVID) v6.8 [21]. GO terms and KEGG pathways with adjusted *P*-value below 0.05 were considered as significantly enriched by DEGs. The volcano map was drawn by R language with ggplot2. The bar and bubble graphs are plotted by the GOplot package in R. The network graph of cancer related pathways with DEGs was produced by Cytoscape 3.7.1 [22].

### Transcription Factor Analysis

The target genes of *MYC* analyzed in this study were found by Gene Transcription Regulation Database (GTRD) [23] and Database of Human Transcription Factor Targets (hTFtarget) [24]. We found *MYC* target genes from GTRD in *Homo sapiens* with the promoter setting from − 1000 to + 100. And we got target genes of *MYC* from hTFtarget with the default mode. Then we took the intersection from the above two lists for further analysis.

## Result

### Differential Expression Analysis of Eucalyptol Treatment

**Fig. 1:**
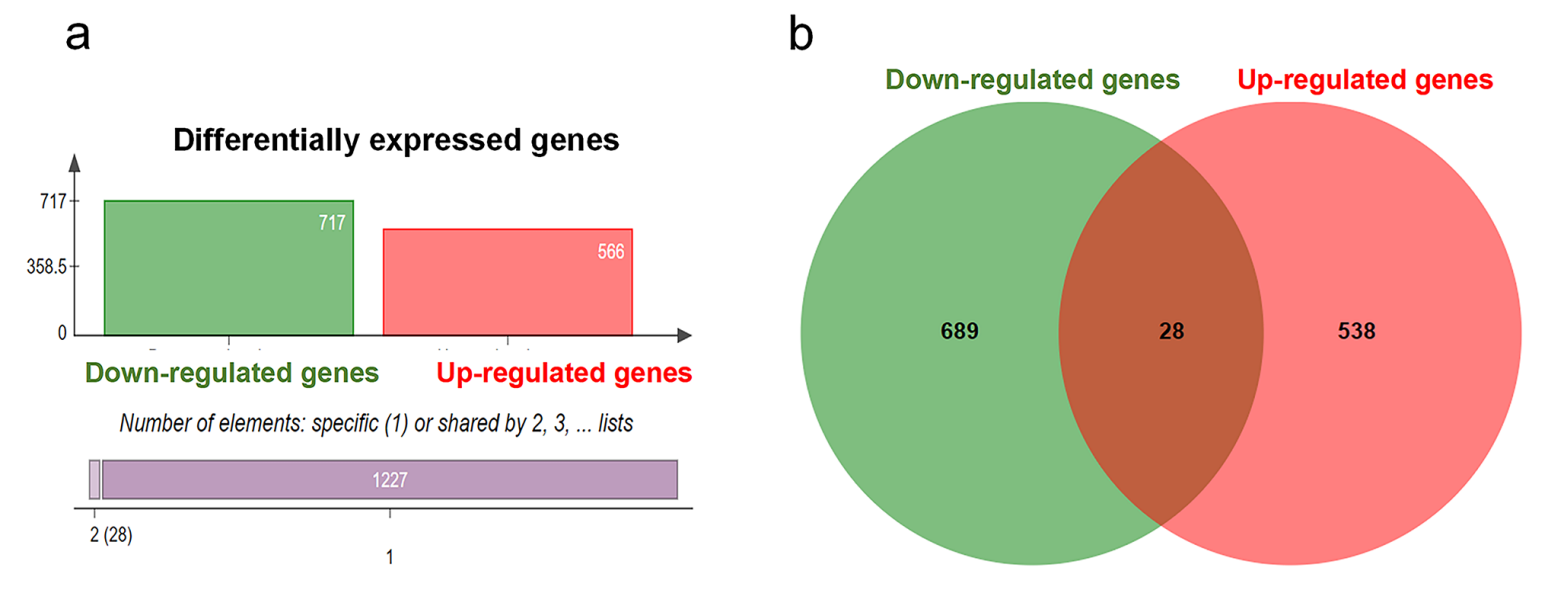
Differentially expression genes of eucalyptol treated SH-SY5Y. a shows the classification of differentially expressed genes by bar graphs, b is the Venn Diagram of down-regulated genes and up-regulated genes

**Fig. 2:**
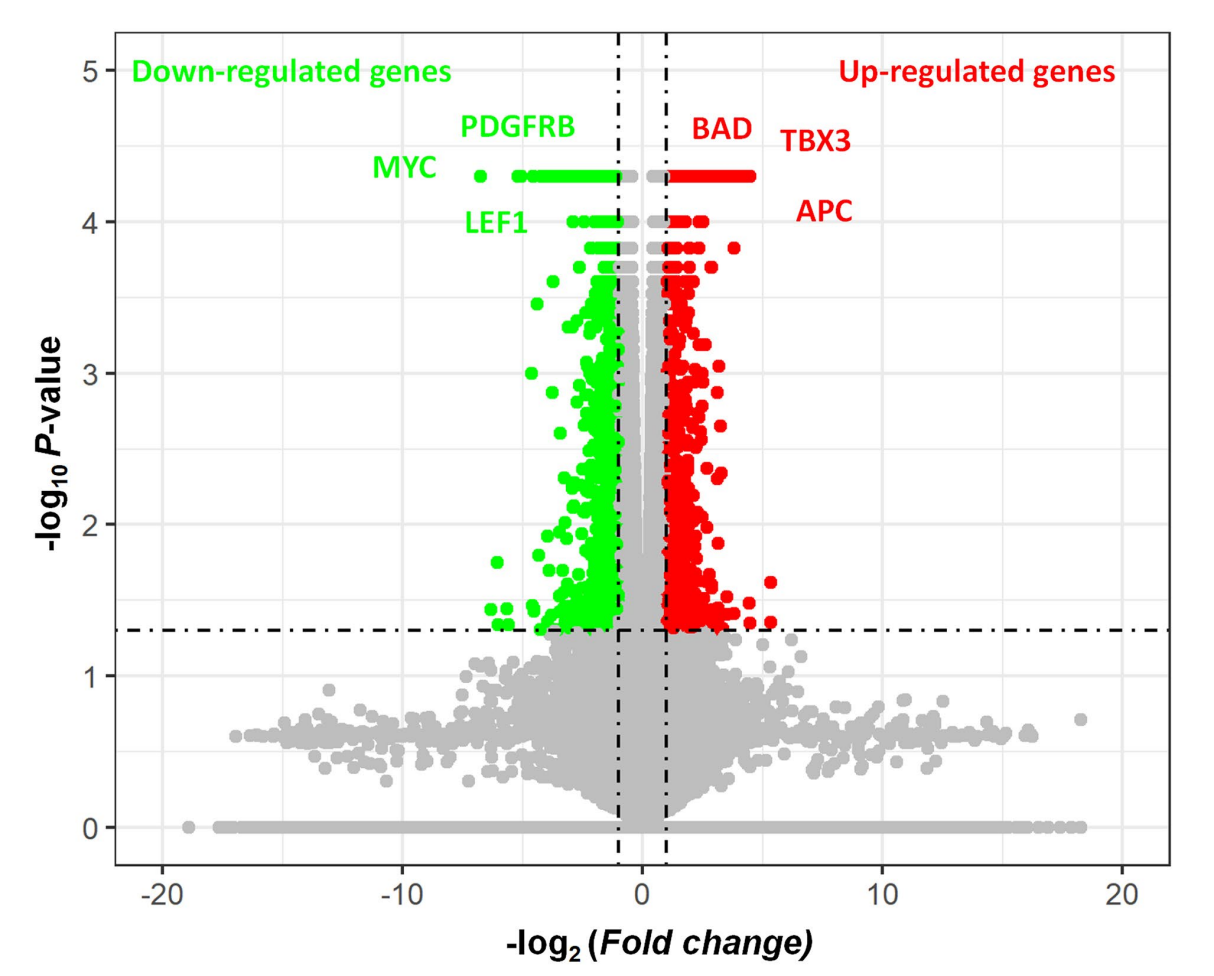
Volcano map of gene expression changing after eucalyptol treatment. Red dots represent up-regulated genes. Green dots represent down-regulated genes

In order to identify the DEGs (up-regulated and down-regulated expression) in SH-SY5Y cells after eucalyptol treatment, we performed mRNA sequencing of normal SH-SY5Y and 100 µM treated SH-SY5Y on the 6th day. RNA-seq identification of DEGs was measured by TopHat and Cufflinks (See Methods). As the results shown in Fig. 1, a total of 1255 genes (1350 transcripts) were differentially expressed, including 566 up-regulated genes (593 transcripts) and 717 down-regulated genes (757 transcripts). There were 28 DEGs with both up-regulated transcripts and down-regulated transcripts. It can be shown from volcano map that anti-tumor genes *BAD3*, *TBX3* and *APC* were up-regulated genes, at the same time, oncogenes *LEF1*, PDGFRB, and *MYC* were down-regulated genes (Fig. 2).

### GO Enrichment Analysis Identified the Biological Functions of DEGs in SH-SY5Y After Eucalyptol Treatment

**Fig. 3:**
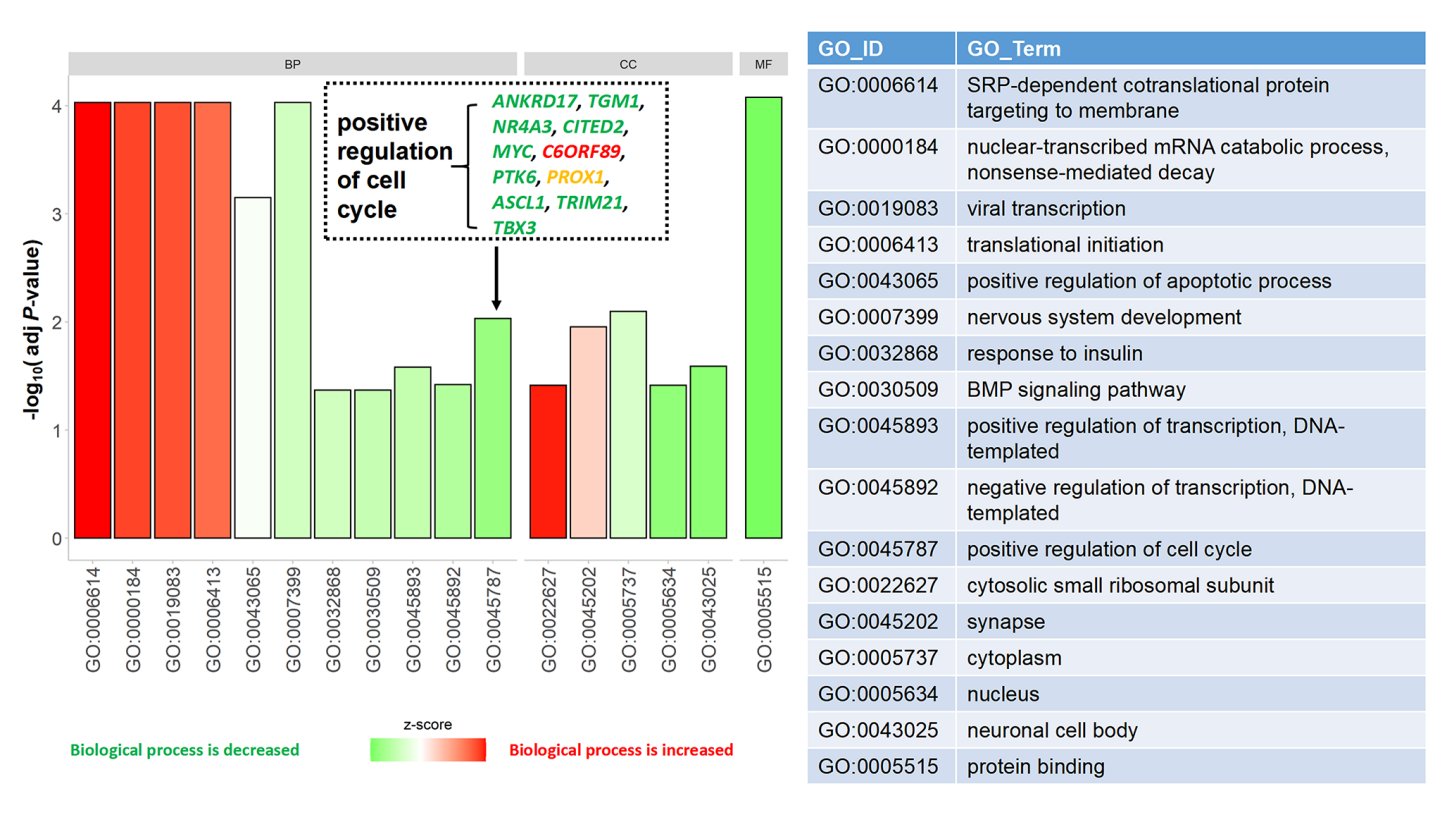
Z-score coloured barplot of GO enrichment of DEGs. If the biological process (/molecular function/cellular components) is decreased, the colour of the bar is green. And If the biological process (/molecular function/cellular components) is increased, the colour of the bar is red

To further evaluate the biological functions of these DEGs, GO enrichment analysis was performed on the experimental group. The results revealed that there was significant enrichment of GO terms, which are grouped into three categories: molecular function (MF), cellular component (CC) and biological process (BP). In the biological process, GO: 0007399: nervous system development, GO: 0000184: nuclear-transcribed mRNA catabolic process, nonsense-mediated decay, GO: 0006614: SRP-dependent cotranslational protein targeting to membrane, GO: 0006413: translational initiation, GO: 0019083: viral transcription, GO: 0043065: positive regulation of apoptotic process, GO: 0045787: positive regulation of cell cycle, GO: 0045893: positive regulation of transcription, DNA-templated, GO: 0045892: negative regulation of transcription, DNA-templated, GO:0032868: response to insulin, GO: 0030509: BMP signaling pathway had the most abundant GO function items. In the molecular function, GO: 0005515: protein binding had the most abundant GO function items. In the cellular component, GO: 0005737: cytoplasm, GO: 0045202: synapse, GO: 0043025: neuronal cell body, GO: 0022627: cytosolic small ribosomal subunit, GO: 0005634: nucleus (Fig. 3).

It should be mentioned that GO: 0045787 (positive regulation of cell cycle) was negatively regulated for the most involved DEGs in this term were down-regulated. Those decreasing DEGs were *ANKRD17*, *TGM1*, *NR4A3*, *CITED2*, *MYC*, *PTK6*, *ASCL1*, *TRIM21*, *TBX3*.

### KEGG Enrichment Analysis Identified Cancer Related Pathways

**Fig. 4:**
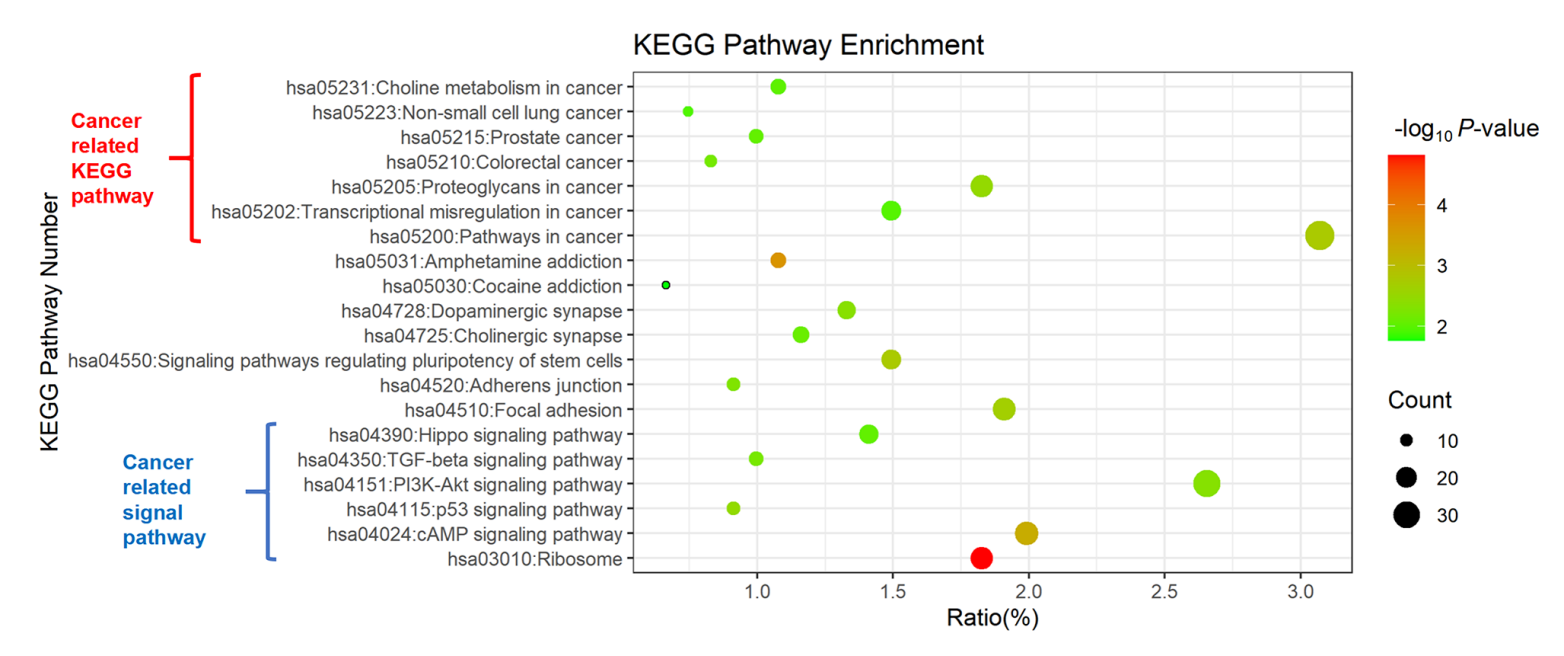
Bubble map of KEGG pathway enrichment of DEGs. The size of bubbles represents the count of DEGs. The color of bubbles represents *P*-value

To analyse the DEGs, KEGG enrichment analysis was used for annotation. The top 20 enrichment KEGG pathways, including hsa03010: Ribosome, hsa05031: Amphetamine addiction, hsa04024: cAMP signaling pathway, hsa05200: Pathways in cancer, hsa04550: Signaling pathways regulating pluripotency of stem cells, hsa04510: Focal adhesion, hsa05205: Proteoglycans in cancer, hsa04115: p53 signaling pathway, hsa04728: Dopaminergic synapse, hsa04151: PI3K-Akt signaling pathway, hsa04520: Adherens junction, hsa04350: TGF-beta signaling pathway, hsa05210: Colorectal cancer, hsa04725: Cholinergic synapse, hsa05215: Prostate cancer, hsa04390: Hippo signaling pathway, hsa05231: Choline metabolism in cancer, hsa05202: Transcriptional misregulation in cancer, hsa05223: Non-small cell lung cancer, hsa05030: Cocaine addiction, were shown in Fig. 4.

The results demonstrated that the seven enriched pathways were directly cancer-related KEGG pathways, as shown in the top 7 pathways in Fig. 4. We also found that five cancer-related cellular signaling pathways, including Hippo signaling pathways, TGF-beta signaling pathways, PI3K-Akt signaling pathway, p53 signaling pathway, cAMP signaling pathway. These results suggest that eucalyptol may exert antitumor effects through the above signaling pathways.

### System Biological Analysis Identified the Key Genes in the Network of Cancer Related Pathways with DEGs

**Fig. 5:**
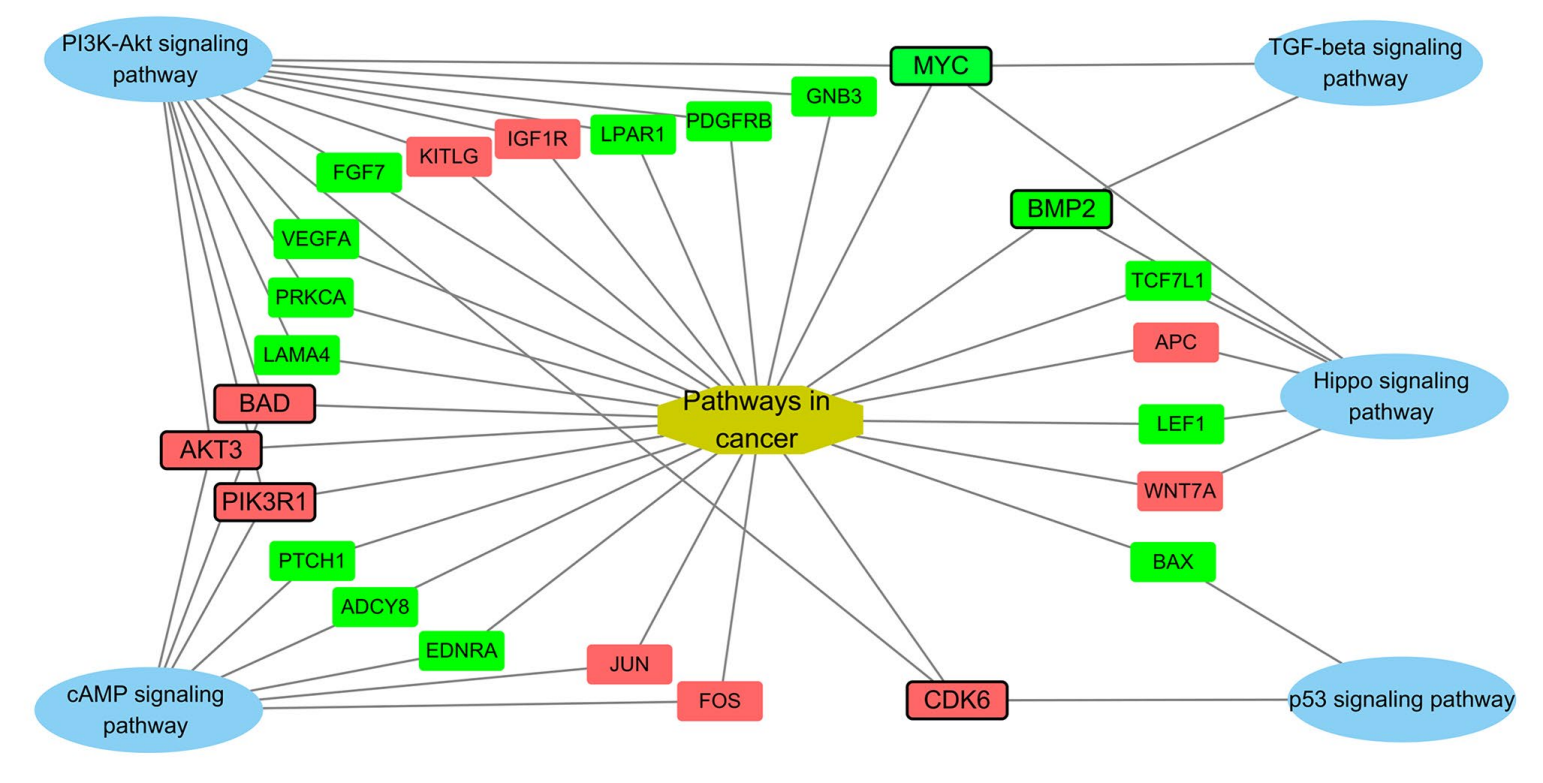
The network of cancer-related KEGG pathways with DEGs. The up-regulated genes are in red boxes, while down-regulated genes are in green boxes

For clarify the mechanism of anti-proliferation, we constructed a network of the KEGG enriched cellular signaling pathways (Hippo signaling pathways, TGF-beta signaling pathways, PI3K-Akt signaling pathway, p53 signaling pathway, cAMP signaling pathway) and Pathways in cancer with DEGs (Fig. 5). We found that *MYC*, *BMP2*, *CDK6*, *PIK3R1*, *AKT3* and *BAD* are linked with more than 3 pathways, demonstrating those genes are important roles in anti-proliferation effect of eucalyptol on SH-SY5Y.

### Transcription Factor Analysis Showed Multiple Biology Functions of *MYC* in the Antitumor Mechanism of Eucalyptol

**Fig. 6:**
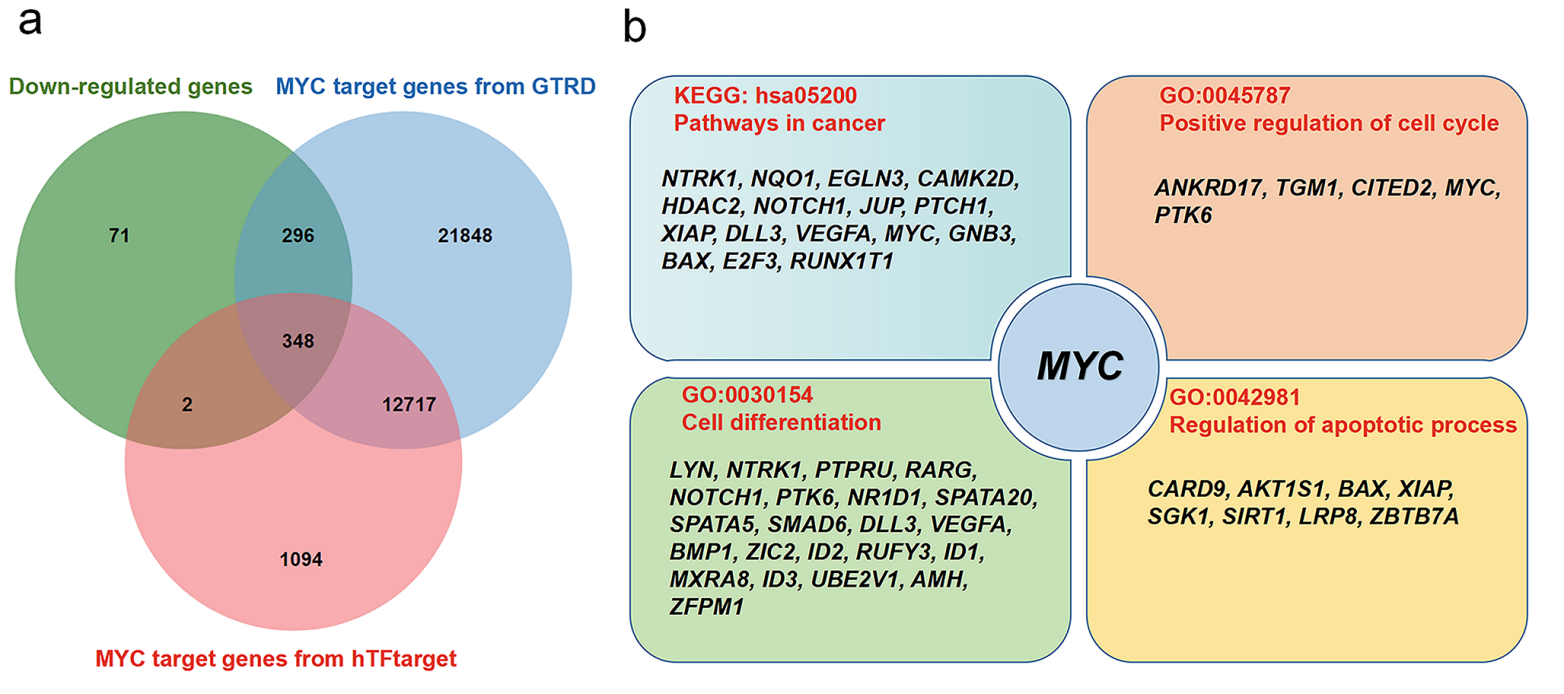
Transcription factor analysis of *MYC* target gene. a shows the Venn Diagram of the list of down-regulated genes with the list of these two *MYC* target gene lists from GTRD and hTFtarget. b shows MYC targets genes of cancer related pathways by the KEGG and GO enrichment

*MYC* is an important cancer-related transcription factor gene and sits on the most important gene node in the network (Fig. 5), suggesting that it plays an important role in the antitumor mechanism of eucalyptol. In this study, it was found that eucalyptol caused a pronounced down-regulation of *MYC* expression and then might result in the down-regulation of *MYC* target genes (MTGs). Therefore, we conducted a transcription factor analysis on *MYC*. First, we found 35,769 MTGs from GTRD and 14,741 MTGs from hTFtarget. Then we intersected the list of down-regulated genes with the list of these two MTG lists to determine the down-regulated genes regulated by *MYC* (Fig. 6a). After analysis, we found that about half of the down-regulated genes were *MYC* target genes (48.5%, 348/717). By KEGG and GO analysis of these MTGs, we found that these genes were enriched in KEGG: HSA05200 Pathways in cancer, GO:0045787 Positive regulation of cell cycle, GO:0030154 Cell differentiation, GO:0042981 regulates the passage of apoptotic process (Fig. 6b).

## Discussion

Moxibustion is an effective supportive cancer care in inhibiting tumor growth [25, 26] and alleviating side effects of chemotherapy and radiotherapy [27]. Such as, moxibustion can inhibit nausea and vomiting after chemotherapy [28, 29]. In addition, moxibustion can also be used to treat cancer-related fatigue [30]. The mechanism of moxibustion therapy for cancer is still unclear. Eucalyptol is the main component of *Artemisia argyi* [11, 12]. Eucalyptol has been reported to inhibit the proliferation of many cancer cells [22, 23, 31–33]. In this study, we revealed the anti-tumor effect and mechanism of eucalyptol against human neuroblastoma SH-SY5Y cells by transcriptome sequencing.

The mechanism of the anti-tumor effect of eucalyptol is complex and has not been fully clarified. Suppression of growth by eucalyptol in leukemia, ovarian cancer cells and colorectal cell lines was reported to the induction of apoptosis [21–23, 32]. It was reported that eucalyptol also inhibited cell proliferation by promoting G0/G1 arrest in HepG2 cells [32]. In our study, we found that eucalyptol has a negative effect on “positive regulation of cell cycle (GO: 0045787)” by reducing the expression of most genes in this GO terms (Fig. 3), indicating that eucalyptol intervene cancer cell growth not only by inducing apoptosis but also with anti-proliferation.

Cancer is a complex disease characterized by excessive proliferation of cancer cells with selective growth advantage [34]. Many cell signaling pathways related to cancer development, such as PI3K-Akt signaling pathway [35, 36], Ras signaling pathway [37–40], STAT signaling [40–43], MAPK signaling pathway [35, 37], TGF-beta signaling pathway [44, 45], NOTCH signaling pathway [46–49], p53 signaling pathway [50–52], cAMP signaling pathway [53, 54], Hippo signaling pathway [55, 56], Wnt signaling pathway [57], and so on. In our study, we found that some cancer-related signaling pathways were enriched based on DEGs, including Hippo signaling pathways, TGF-beta signaling pathways, PI3K-Akt signaling pathway, p53 signaling pathway, cAMP signaling pathway. This result suggests that eucalyptol can regulate multiple cancer-related signaling pathways to achieve its anti-cancer effect.

With system biological analysis of the network constructed by cancer related pathways and DEGs, we found that *MYC* is a key gene in the network of eucalyptol’s anti-tumor mechanism. *MYC* is an important and well-known oncogene, which regulates cell growth and proliferation [58, 59]. A previous study reported that eucalyptol inhibited protein expression of *MYC* in AGE-treated podocytes and diabetic kidneys [60]. In our study, we found eucalyptol can down-regulated *MYC* transcription in neuroblastoma SH-SY5Y cell. And half of the down-regulated genes were *MYC* target genes. Some of those genes are related to positive regulation of cell cycle, cell differentiation, and apoptotic process, indicating that eucalyptol may be implicated in its anti-tumor effects by down-regulating *MYC* and its target genes involved in cell division, differentiation, and apoptosis pathways.

In conclusion, our findings demonstrate that eucalyptol can exert its anti-tumor activity by regulating multiple cancer-related cellular signal pathways in human SH-SY5Y cells *in vitro*. Eucalyptol shows promise as an effective and safe therapeutic agent for neuroblastoma.

## Data Availability

The original data presented in the study are included in the article materials, further inquiries can be directed to the author/corresponding authors.
