# Correction to: Transcriptome Analysis Reveals the Anti-Tumor Mechanism of Eucalyptol Treatment on Neuroblastoma Cell Line SH-SY5Y

**DOI:** 10.1007/s11064-022-03823-6

**Published:** 2022-11-21

**Authors:** Kai Gao, Congying Wu, Yanlong Li, Jian Lu, Yuwu Jiang

**Affiliations:** 1 Department of Pediatrics, Peking University First Hospital, No.1 Xi’ a Men Street, West District 100034 Beijing, China; 2 Department of acupuncture and moxibustion, Dongzhimen Hospital, Beijing University of Chinese Medicine, No.116 Cuiping West Street, Tongzhou District 101121 Beijing, China

**Correction to: Neurochemical Research.**

10.1007/s11064-022-03786-8.

In the original version of the article, unfortunately the typesetting team was missed to incorporate the article note “Kai Gao and Congying Wu contributed equally”. This has been corrected by publishing this correction article. The original version has been updated.

